# Whole-cell circular dichroism difference spectroscopy reveals an *in vivo*-specific deca-heme conformation in bacterial surface cytochromes†
†Electronic supplementary information (ESI) available: Experimental section, supporting figures S1–S14. See DOI: 10.1039/c8cc06309e
‡
‡Y. T. and A. O. designed the study, and Y. T., P. C. and S. H. performed the spectroscopic experiments. Y. T. conducted the electrochemical experiments, L. S. and T. A. C. purified the MtrC, and S. H. and K. I. conducted the theoretical calculations regarding the CD spectra. Y. T., S. H., K. H., K. I., and A. O. prepared the manuscript.


**DOI:** 10.1039/c8cc06309e

**Published:** 2018-11-07

**Authors:** Yoshihide Tokunou, Punthira Chinotaikul, Shingo Hattori, Thomas A. Clarke, Liang Shi, Kazuhito Hashimoto, Kazuyuki Ishii, Akihiro Okamoto

**Affiliations:** a Department of Applied Chemistry , The University of Tokyo , 7-3-1 Hongo , Bunkyo-ku , Tokyo 113-8656 , Japan; b Institute of Industrial Science , The University of Tokyo , 4-6-1 Komaba , Meguro-ku , Tokyo 153-8605 , Japan; c Centre for Molecular and Structural Biochemistry , School of Biological Sciences and School of Chemistry , University of East Anglia , Norwich NR4 7TJ , UK; d Department of Biological Sciences , School of Environmental Studies , The China University of Geosciences , Wuhan , Hubei 430074 , P. R. China; e International Center for Materials Nanoarchitectonics , National Institute for Materials Science , 1-1 Namiki , Tsukuba , Ibaraki 305-0044 , Japan . Email: okamoto.akihiro@nims.go.jp

## Abstract

We established whole-cell circular dichroism difference spectroscopy to identify the inter-heme interaction in deca-heme cytochrome protein MtrC in whole cell. Our data showed that the heme alignment of reduced MtrC in whole cell is distinct from that in purified one, suggesting the *in vivo* specific electron transport kinetics.

## Abstract 2

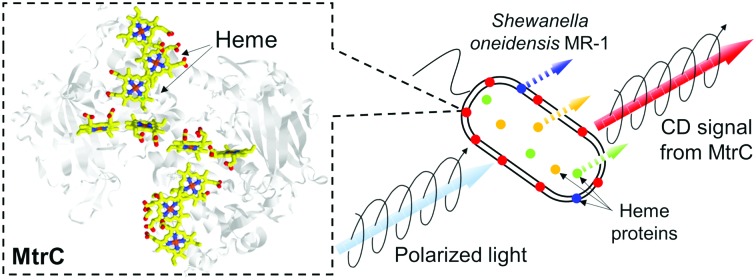
Our novel analytical framework to identify the inter-heme interaction in deca-heme cytochrome protein MtrC in whole cell revealed that the heme alignment in reduced MtrC is distinct from that in purified system.

## 

Bacterial electron transport to a solid substrate or electrode located extracellularly is accomplished by unidirectional electron flow *via* an array of more than twenty heme redox centers arranged in the outer membrane *c*-type cytochrome complex (OM *c*-Cyts).^1^–^4^ The biological electron conduction occurs with a rate constant of 10^4^–10^5^ electrons per second along a distance over 100 Å.^4^–^6^ The ability of the multi-heme alignment and interaction to promote highly efficient long-range electron transport under non-equilibrium conditions has been a focal point for nanoscale electronic applications.^7^,^8^ Recent studies have resolved the three dimensional structures of some units in OM *c*-Cyts, MtrC, MtrF and OmcA, at atomic resolution in a model bacterium, *Shewanella oneidensis* MR-1.^5^,^9^,^10^ The redox potential and electronic coupling among hemes have been investigated using the crystal structure, and significant contributions elucidating functions including electron transfer rates and pathways among hemes have been achieved.^6^,^9^–^13^ However, the structural flexibility of a unit in OM *c*-Cyts has been suggested by small-angle X-ray scattering;^14^ thus, rearrangement of the heme conformation *in vivo* is likely influenced by interactions with the OM, solid electron acceptors, cofactors, and proteins as well as by a shift in equilibrium from the continuous electron flow in a thermodynamically open living system.^15^–^18^ Therefore, establishing a methodology to directly monitor the heme arrangement in intact cells is critical.

The inter-heme conformation and interactions have been studied using circular dichroism (CD) spectroscopy of purified peptides and proteins.^19^–^21^ Given that the ten hemes with a minimal distance of 4 Å in MtrC (Fig. 1 inset) would provide a large amplitude in CD signal according to their exciton coupling between π-conjugated systems, which is inversely proportional to cube of the distance,^9^,^19^,^22^,^23^ the application of *S. oneidensis* MR-1 cells to CD spectroscopy might enable the direct observation of heme conformation in native MtrC. However, direct observation of heme protein encoded by single gene in an intact cell remains a challenge, often due to the presence of other heme proteins. Thus, the abundance of heme-containing genes makes the characterization of MtrC particularly difficult in an intact cell of *S. oneidensis* MR-1.^24^ Here, we established the whole-cell CD difference spectroscopy using *S. oneidensis* MR-1 wild type (WT) and mutant strain lacking MtrC to acquire the CD signal of MtrC under native conditions. Our data revealed that, compared to purified MtrC, reduced, but not oxidized, MtrC in intact cells exhibits a distinct heme alignment and that this alignment likely affects the rate of electron transport.

**Fig. 1:**
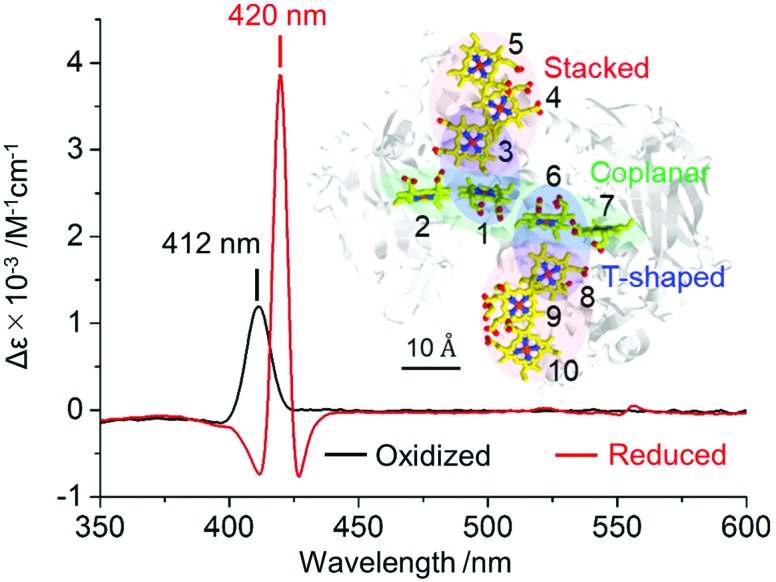
Circular dichroism (CD) spectra of purified MtrC in the visible range. Reduced MtrC is indicated by the red line and was achieved by the addition of 0.67 mM Na_2_S_2_O_4_ to oxidized MtrC, which is depicted by the black line. Inset: Crystal structure of the MtrC protein highlighting the deca-hemes (PDB code: ; 4LM8).^9^ The heme numbers and heme pair motifs are indicated.

First, we examined the extent of exciton coupling among the ten heme centers in MtrC using CD spectroscopy. Purified MtrC in HEPES buffer was added to a Pyrex cuvette with a 1.0 cm optical path length and exhibited a positive peak at 412 nm in the CD spectrum and Soret peak absorption at 410 nm, which was assigned to the hemes in MtrC (Fig. 1 and Fig. S1, ESI†).^25^ Upon MtrC reduction by 0.67 mM Na_2_S_2_O_4_, the positive CD peak signal shifted to 420 nm, and relatively small negative signals appeared (Fig. 1).^22^,^23^ The peak CD signal intensities in the oxidized and reduced MtrC (Δ*ε*) were 1.19 × 10^3^ and 3.86 × 10^3^ M^–1^ cm^–1^, respectively, which are two orders of magnitude larger than that of mono-heme horse heart cytochrome *c* (approximately 17 M^–1^ cm^–1^).^26^ Considering that this intensity is even larger than that of the artificially synthesized bis-porphyrin compound (Δ*ε* is approximately 400 M^–1^ cm^–1^),^22^,^27^,^28^ the Δ*ε* of MtrC is extraordinary large. CD calculations based on the exciton chirality method^19^,^29^ reproduced the relative intensity between reduced and oxidized MtrC (Fig. S2, ESI†), suggesting that the exciton coupling among ten heme centers in MtrC dominates the observed CD intensity. This extraordinary large CD intensity possibly enables CD spectroscopy of an intact cell to reveal the conformation of native MtrC.

However, to accomplish the identification of the CD signal of the MtrC protein in intact *S. oneidensis* MR-1 cells, we had to overcome two problems concerning background signal: light scattering and interference from other cytochrome proteins. Because use of intact cells decreases the detectable light due to scattering from cell surfaces,^16^ the signal-to-noise ratio was poor, and a broad background peak was observed throughout the visible light region (Fig. 2a and Fig. S3, ESI†). Thus, we optimized CD measurement conditions as follows: the cell density was set as an optical density (OD) at 600 nm of 1.33 ± 0.02, and the 1.0 nm bandwidth for purified MtrC was changed to 5.0 nm for intact cells (Fig. S4, ESI†). Second, we minimized interference from the other cytochromes in *S. oneidensis* MR-1 cells by subtracting the CD spectrum from a mutant with a deletion in the gene encoding the MtrC protein (Δ*mtrC*) from the WT spectrum. The CD spectrum of WT suspended in defined-medium in a Pyrex cuvette with a 1.0 cm path length showed a strong signal near the Soret band, which was clearly diminished in Δ*mtrC* (Fig. 2a). The difference in the CD spectrum between WT and Δ*mtrC* showed a large signal with a peak at 413 nm, which is almost identical in wavelength to the purified MtrC protein (Fig. 2b). Furthermore, the reduction of native MtrC in *S. oneidensis* MR-1 by the addition of an electron donor, 30 mM lactate, under anaerobic condition shifted the peak to a longer wavelength centered at 421 nm with splitting signals (Fig. 2b and Fig. S5, ESI†). The peak position and peak width in both the oxidized and reduced states were almost identical to the CD spectrum of the purified MtrC protein (Fig. 2b).

**Fig. 2:**
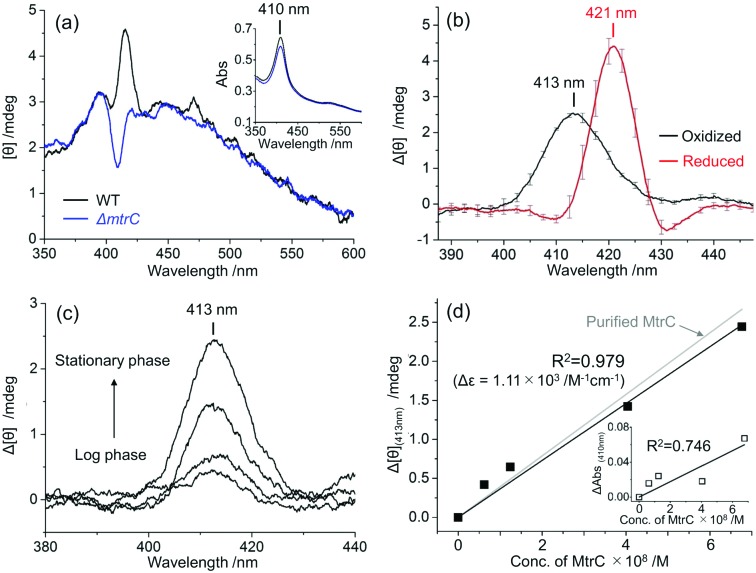
(a) Circular dichroism (CD) spectra of whole *S. oneidensis* MR-1 cells (wild-type, WT; black line) and a mutant strain deficient for the *mtrC* gene (Δ*mtrC*; blue line). The cell density was adjusted to OD_600_ = 1.33 ± 0.02. Inset: Absorption spectra of the same samples in diffused transmission mode. (b) Differences in CD spectra between WT and Δ*mtrC* oxidized by oxygen (black line) and reduced by 30 mM lactate (red line). The error bars represent the mean ± SEM obtained using three individual samples and eight measurements each. (c) The differences in CD spectra between WT and Δ*mtrC* at various growth phases in the oxidized state. (d) Plots of the changes in CD signal intensity at 413 nm observed in (c) (Δ[*θ*]_(413nm)_) against the MtrC concentration in the cell suspension. The squares of the correlation coefficients (*R*^2^ = 0.979, black line) include the point of origin. The gray line represents the CD signal intensity for purified MtrC at 412 nm estimated from Δ*ε* = 1.19 × 10^3^ M^–1^ cm^–1^. Inset: The plots of ΔAbs at 410 nm between WT and Δ*mtrC* against the MtrC concentration.

Correlation of the intensity of the Soret CD peak with the concentration of the MtrC protein in cell suspension confirmed the assignment of the CD peak signals as native MtrC. Fig. 2c and d show the relationship between the intensity of the whole-cell CD difference spectrum at 413 nm (Δ[*θ*]_(413nm)_) and the amount of MtrC in the cell suspension during various aerobic growth phases estimated by SDS-PAGE.^30^,^31^ Consistent with a previous report,^32^ the MtrC concentration in the cell suspension increased depending on the growth phase and the deletion of *mtrC* gene had scarce impact on gene expression of other major proteins (Fig. 2c, d and Fig. S6, ESI†). While the Soret absorption peak intensity indicates that the amount of MtrC is less than 20% of total cytochrome proteins (Fig. 2a inset), the Δ[*θ*]_(413nm)_ linearly increased with MtrC concentration, and the squares of the correlation coefficient of 0.979 (black line in Fig. 2d) passed through the origin. This direct positive relationship strongly suggests that the whole-cell CD difference spectrum in the Soret region represents the signal from the native MtrC protein. In contrast, the amounts of other cytochromes in the cell fluctuate in each growth phase, as the absorption peak intensity at 410 nm showed poor correlation with the amount of the MtrC protein (Fig. 2d inset, *R*^2^ = 0.746), further supporting that the Soret CD signal is specific for the MtrC protein in native environment.

Notably, the Soret CD peak intensity of oxidized native MtrC obtained from the slope in Fig. 2d (Δ*ε*_(413nm)_ = 1.11 × 10^3^ M^–1^ cm^–1^) was almost identical to that of purified MtrC (Δ*ε*_(412nm)_ = 1.19 × 10^3^ M^–1^ cm^–1^), indicating that native MtrC maintains the arrangement of heme centers. In contrast, once native MtrC was reduced, the Soret CD intensity (whole-cell: Δ*ε* = 1.97 × 10^3^ M^–1^ cm^–1^) was approximately twofold lower than that of purified MtrC (Δ*ε* = 3.86 × 10^3^ M^–1^ cm^–1^), and the splitting signals around the positive peak at 420 nm were suppressed (Fig. 3a and Fig. S7, ESI†), indicating the possibility of a conformational change in native MtrC associated with the reduction reaction. Furthermore, purified MtrC exhibited a Soret CD signal with a distinct pH dependency in the reduced state (Fig. 3b and Fig. S8, ESI†). In the oxidized state, both purified and native MtrC maintained the Soret peak at a similar intensity throughout the physiological pH range. However, reduced native MtrC exhibited a drastic decrease in Δ*ε* of Soret CD peak at a pH of approximately 7 that was not observed in purified MtrC (Fig. 3b and Fig. S8–S10, ESI†). These distinct CD profiles suggest that the inter-heme interaction of reduced MtrC differs between in native and purified system. In addition to inter-heme interaction, aromatic amino acids in the vicinity of hemes, specifically bis-histidine coordinated to deca-hemes, potentially alter CD amplitude.^33^,^34^ Therefore, we conducted magnetic CD (MCD) spectroscopy to examine the coordination state of heme centers in reduced native and purified MtrCs. Both MCD spectra exhibited a dispersion-type Faraday A term with a center wavelength of approximately 550 nm (Fig. S11, ESI†), which is characteristic of the low-spin Fe^2+^ state. These data suggest that the coordination of bis-histidine with the deca-hemes is maintained inside native MtrC as well as purified MtrC in the reduced state^35^–^37^ and has little effect on Soret CD peak intensity. Therefore, the CD signal decrease in reduced MtrC in native compared with purified system (Fig. 3a) and at a pH of approximately 7 in native reduced MtrC (Fig. 3b) are likely assignable to changes in inter-heme interaction.

**Fig. 3:**
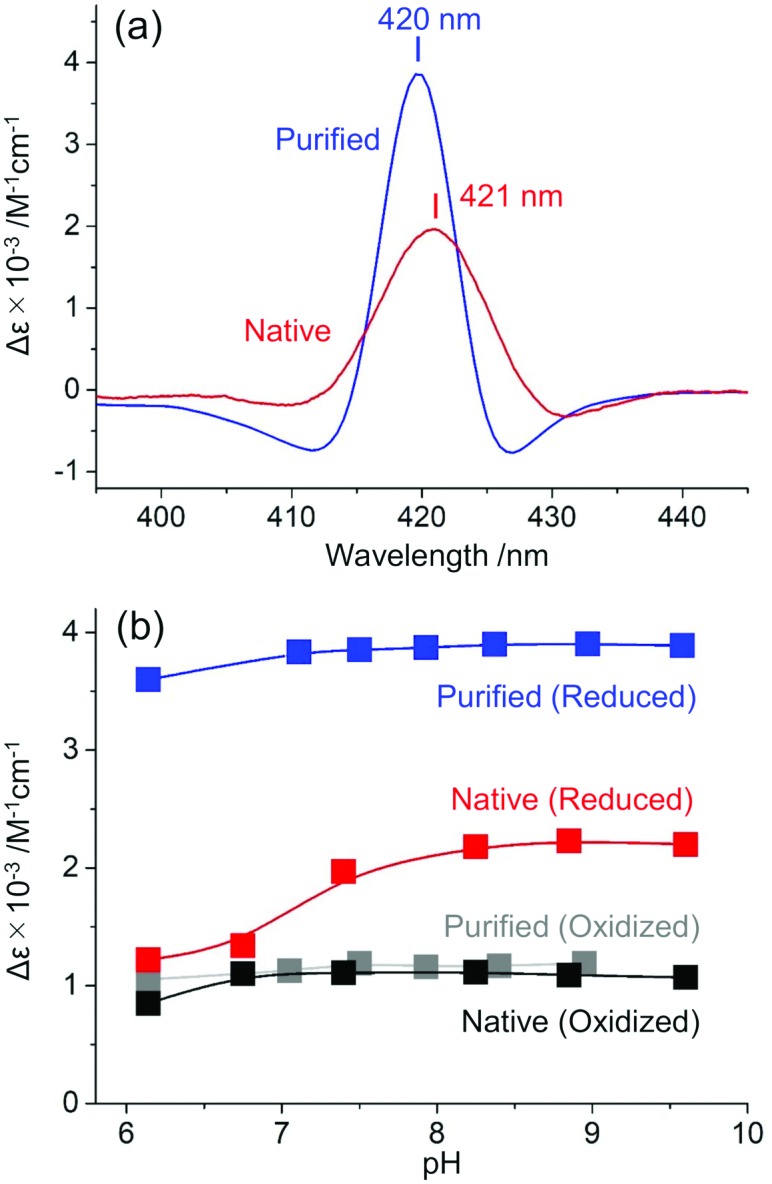
(a) Circular dichroism (CD) spectra of purified MtrC (blue line) and native MtrC in whole cells (red line) in the reduced state at pH values of 7.5 and 7.4, respectively. The Δ*ε*_(420nm)_ for purified MtrC and the Δ*ε*_(421nm)_ for native MtrC are 3.86 × 10^3^ M^–1^ cm^–1^ and 1.97 × 10^3^ M^–1^ cm^–1^, respectively. (b) Soret peak intensities in the CD spectra of purified reduced MtrC (Δ*ε*_(420nm)_, blue), native reduced MtrC (Δ*ε*_(421nm)_, red), purified oxidized MtrC (Δ*ε*_(412nm)_, gray), and native oxidized MtrC (Δ*ε*_(413nm)_, black) as a function of pH.

Accordingly, the electron transport kinetics through the heme centers in native MtrC represented a similar pH dependency with CD signal. We performed a whole-cell electrochemical assay to probe the microbial lactate-oxidation current, which is limited by the rate of electron transport *via* OM *c*-Cyts under our experimental conditions (Fig. S12, ESI†).^38^,^39^ At a pH below 7, current production at +0.4 V (*versus* a standard hydrogen electrode) significantly decreased compared with higher pH conditions (Fig. S12, ESI†), suggesting that the CD signal decrease at pH of between 7.4 and 6.8 corresponded with the rate of electron transport *via* the heme conduit in MtrC. In contrast, such pH dependency was not observed in the redox property of purified MtrC protein,^25^ which is also consistent with the pH susceptibility of CD signal in purified MtrC (Fig. 3b). Assuming that the conformational change of hemes in reduced native MtrC decreases the electron flux, the critical conformational change may occur in T-shaped heme pairs (1,3 and 6,8) and coplanar heme pairs (2,1, 1,6 and 6,7) (Fig. 1 inset) because they mediate slower electron transport reactions than other stacked heme pairs in MtrF, which is a homologue of MtrC.^6^ Given that the orientation change in T-shaped heme pairs potentially alters the electron transfer matrix element over 1000 times,^40^ T-shaped heme pairs are the most likely candidates causing the decrease of electron transport rate. Similarly, the Δ*ε* calculation based on the exciton chirality method^19^,^29^ indicated that the T-shaped heme pairs have a larger impact on not only electron transport kinetics but also CD signal intensity compared with coplanar pairs (Fig. S13, ESI†).^22^,^27^ However, given the stacked heme pairs provided more significant contribution in Δ*ε* value, the pH dependent conformational change in reduced native MtrC may not be limited to T-shaped heme pairs but also heme conduit as a whole.

Similarly, the large difference in the CD intensity of reduced MtrC between in native and purified system (Fig. 3a) may be associated with changes in interaction of heme conduit including T-shaped pairs, given the native OM *c*-Cyts shows over 10 times lower electron transport kinetics than purified OM *c*-Cyts at a neutral pH.^41^–^43^ Since previous study demonstrated that associated proton transfer limits the rate of current production of *S. oneidensis* MR-1,^38^ heme conformation might also impact the proton transfer kinetics. Considering that the significant suppression in Soret CD signal in native MtrC was diminished in purified MtrCAB complex (Fig. S14, ESI†), the *in vivo*-specific conformational change is potentially caused by unfolding reaction of reduced MtrC assisted by other components of OM *c*-Cyts such as OmcA, other membrane proteins, and/or lipopolysaccharide.^44^ It would be, therefore, interesting to further identify the critical molecular aspects using a combination of whole-cell CD spectroscopy and biochemical techniques such as point mutations.

In this study, we established whole-cell difference CD spectroscopy to investigate inter-heme interactions in native MtrC protein by using wild-type and a mutant strain lacking MtrC in *S. oneidensis* MR-1. Comparisons of MtrCs in purified and native systems suggested that the heme conformation in reduced MtrC is altered in whole cells and that this alteration might critically affect electron transport kinetics. Gaining insight into multi-heme cytochromes in an intact cell by using mutant strains has broad applicability beyond the bacterial species, such as *Geobacter sulfurreducens* PCA.^45^,^46^ We anticipate that the combination of this whole-cell CD spectroscopy method with electrochemistry would enable monitoring of MtrC conformation in a state where the equilibrium is shifted by continuous electron flow in a thermodynamically open system,^15^,^16^ which is fundamental for understanding biological redox reactions.

We thank Prof. Kohei Uosaki and Prof. Hiroyuki Noji for their advice. This work was supported financially by a Grant-in-Aid from the Japan Society for Promotion of Science (JSPS) KAKENHI Grant Number 17H04969 to A. O. and 17J02602 to Y. T., the US Office of Naval Research Global (N62909-17-1-2038) and the Japan Agency for Medical Research and Development (17gm6010002h0002), National Natural Science foundation of China (41630318 & 41772363), and Biotechnology and Biological Sciences Research Council (BB/P01819X). It was also supported by the NIMS Molecule & Material Synthesis Platform in “Nanotechnology Platform Project” operated by the Ministry of Education, Culture, Sports, Science and Technology (MEXT), Japan. Y. T. is a JSPS Research Fellow and supported by JSPS through the Program for Leading Graduate Schools (MERIT).

## Conflicts of interest

The authors declare no competing financial interest.

## Supplementary Material

Supplementary information
