# Supplementary material for: Whole-cell circular dichroism difference spectroscopy reveals an in vivo-specific deca-heme conformation in bacterial surface cytochromes
Source: Chem Commun (Camb). 2018 Nov 7;54(99):13933–6. doi: 10.1039/c8cc06309e (PMC6301274; doi:10.1039/c8cc06309e)
Supplement: Supplementary file 1 [file CC-054-C8CC06309E-s001.pdf]

Electronic Supplementary Material (ESI) for ChemComm.
This journal is © The Royal Society of Chemistry 2018

Supporting Information

Whole-Cell Circular Dichroism Difference Spectroscopy Reveals an *In Vivo*-Specific Deca-Heme Conformation in Bacterial Surface Cytochromes

Yoshihide Tokunou[a], Punthira Chinotaikul[a], Shingo Hattori[b], Thomas A. Clarke[c], Liang Shi[d], Kazuhito Hashimoto[e], Kazuyuki Ishii[b], and Akihiro Okamoto[e]*

[a]Department of Applied Chemistry, The University of Tokyo, 7-3-1 Hongo, Bunkyo-ku, Tokyo 113-8656, Japan.

[b]Institute of Industrial Science, The University of Tokyo, 4-6-1 Komaba, Meguro-ku, Tokyo 153-8605, Japan.

[c]Centre for Molecular and Structural Biochemistry, School of Biological Sciences and School of Chemistry, University of East Anglia, Norwich NR4 7TJ, United Kingdom

[d]Department of Biological Sciences, School of Environmental Studies, The China University of Geosciences, Wuhan, Hubei 430074 P.R. of China.

[e]International Center for Materials Nanoarchitectonics, National Institute for Materials Science, 1-1 Namiki, Tsukuba, Ibaraki 305-0044, Japan.

E-mail: okamoto.akihiro@nims.go.jp

Experimental Section

Preparation of MtrC protein

MtrC was purified as described previously (M. J. Edwards *et al, Sci. Rep.*, **2015**, 5, 11677) (Robert S. Hartshorne *et al, J. Biol. Inorg. Chem.,* **2007**, 12, 1083–1094). The concentration of purified MtrC was determined by UV-vis absorption spectroscopy of the air-equilibrated protein using the extinction coefficient $\varepsilon_{(410nm)} = 1.26\times10^6$ /M^{-1} cm^{-1} (Robert S. Hartshorne *et al, J. Biol. Inorg. Chem.,* **2007**, 12, 1083–1094). Before the spectroscopic measurement, the MtrC solution was subjected to dialysis and pH was kept at 7.5 with 50 mM HEPES ((2-[4-(2-hydroxyethyl)-1-piperazinyl] ethanesulfonic acid) buffer.

Microbial strains and culture conditions

Shewanella oneidensis MR-1 and *Escherichia coli* K12 was aerobically cultured in 15 mL Luria-Bertani [25 gL^{-1}] medium shaking at 160 rpm for 24 hour at 30°C until stationary growth phase. Subsequently, the cell suspension was centrifuged at 6,000 × *g* for 10 min, and the resultant cell pellet was washed twice with 15 mL Defined Medium (15 mM Sodium succinate, 9.0 mM $(NH_4)_2SO_4$, 5.7 mM K_2HPO_4, 3.3 mM KH_2PO_4, 2.0 mM $NaHCO_3$, 1.0 mM $MgSO_4 \cdot 7H_2O$, and 0.49 mM $CaCl_2$; pH 7.4). Unless noted, the cell density was adjusted by optical density at 600 nm (OD_{600}) as 1.33 ± 0.02 just prior to the measurement. Mutant strain deficient in *mtrC* gene was constructed as described previously (A. Okamoto *et al, Electrochim. Acta,* **2011**, 56(16), 5526-5531).

Instrumentation and conditions for CD spectroscopy and MCD spectroscopy

CD spectroscopy was performed on J-1500 (JASCO) CD spectrometer at room temperature (25 ± 1°C). The spectra are collected in a Pyrex cuvette with path length 1.0 cm under following conditions: 500 nm min^{-1} scan rate, 0.1 nm data pitch (0.5 nm data pitch for purified MtrC), and 5.0 nm bandwidth (1.0 nm bandwidth for purified MtrC). The cell suspension was incubated for 10 minutes in the presence of oxygen or 30 mM lactate prior to spectral analysis to equilibriate the native MtrC to be oxidized or reduced state, respectively. No spectral change was confirmed over 30 minutes incubation. Unless noted, four and eight spectra were integrated for purified MtrC protein and bacterial cells, respectively. MCD spectroscopy was performed on E-250 (JASCO) spectrometer equipped with a JASCO electromagnet (+1.3~-1.3 T) using the same cuvette with CD spectroscopy under the condition of 600 nm min^{-1} scan rate, 0.5 nm data pitch, and 6.0 nm bandwidth (1.0 nm bandwidth for purified MtrC) at room temperature (25 ± 1°C).

Quantification of MtrC in *S. oneidensis* MR-1 by CD spectroscopy and sodium dodecyl sulfate-polyacrylamide gel electrophoresis (SDSPAGE)

S. oneidensis MR-1 and Δ*mtrC* were aerobically cultured in 200 mL bottle containing 100 mL Luria-Bertani (LB; 25 gL^{-1}) medium shaking at 160 rpm at 30°C and sampled about 15 mL at some timings during growth. The obtained cell suspension was washed twice with 10 mL defined medium. The half of the cell was applied to CD spectroscopy and the other was subjected to disruption by CelLytic B (Sigma-Aldrich) followed by quantification of MtrC in heme-dependent SDSPAGE. In detail, 400 µL of CelLytic B was added to the pellet made of 1.0 mL cell suspension of OD_{600} = 1.0 and the solution was gently mixed. After 15 minutes, the solution was diluted to appropriate concentration by sample buffer and applied to SDSPAGE as described previously (Thomas P. E. *et al, Anal. Biochem.,* **1976**, 75(1), 168-176). Since the band intensity increases with the amount of heme (Goodhew C. F. *et al, Biochim. Biophys. Acta,* **1986**, 852(2-3), 288-294), we estimated the amount of MtrC from the band intensity measured by free-software, Image J, using the band from horse heart cytochrome *c* as standard curve. The position of band corresponding MtrC protein was confirmed by purification of MtrCAB complex as reported (Daniel E. Ross *et al, Appl. Environ. Microbiol.,* **2007**, 73(18), 5797-5808).

Electrochemical measurements of current generation from *S. oneidensis* MR-1

Electrochemical experiments were conducted in a single chamber, three-electrode system, as described in previous reports (Y. Tokunou, *et al, J. Vis. Exp.*, **2018**, 134, 57584) (A. Okamoto *et al, Electrochim. Acta,* **2011**, 56(16), 5526-5531) (Y. Tokunou *et al, J. Phys. Chem. C*, **2016**, 120(29), 16168-16173). The three-electrode system comprised of an indium tin-doped oxide (ITO) substrate (surface area of 3.1 cm^2) as the working electrode at the bottom of the chamber, and Ag/AgCl (KCl saturated) and a platinum wire, which were used as reference and counter electrodes, respectively. *S. oneidensis* MR-1 cells aerobically cultured in 15 mL Luria-Bertani [25 gL^{-1}] medium for 24 hour at 30°C was centrifuged at 6,000 × *g* for 10 min, and the resultant cell pellet was resuspended in 15 mL of electrochemical medium (pH 7.8) (EM: $NaHCO_3$ [2.5 g], $CaCl_2{\cdot}2H_2O$ [0.08 g], NH_4Cl [1.0 g], $MgCl_2{\cdot}6H_2O$ [0.2 g], NaCl [10 g], yeast extract [0.5 g], and (2-[4-(2-hydroxyethyl)-1-piperazinyl] ethanesulfonic acid [HEPES; 7.2 g] [per liter]) supplemented with 10 mM lactate as the source of carbon for *S. oneidensis* MR-1. The cells were further cultivated aerobically at 30°C for 12 hours and centrifuged again at 6,000 × *g* for 10 min. The resultant cell pellet was then washed twice with EM medium by centrifugation for 10 min at 6,000 × *g* before electrochemical experiment. Four milliliters of EM containing lactate (10 mM), where pH was adjusted by NaOH or H_2SO_4, was deaerated by bubbling with N_2 and added into the electrochemical cell as an electrolyte. The reactor was maintained at a temperature of 303 K and was not agitated during the measurements. A cell suspension of *S. oneidensis* MR-1 in EM with OD_{600} of 0.1 was injected into the electrochemical cell with a potential of +0.4 V versus standard hydrogen electrode (SHE).

CD calculation based on the exciton chirality method

CD calculations based on the exciton chirality method were carried out in both oxidized and reduced MtrC. To calculate the CD spectra, the excited-state energies (ε) and the rotational strengths (R) were evaluated by a diagonalization of a determinant, in which off-diagonal terms (V_{ijkl}) were obtained by a point-dipole approximation. Here, electric transition dipole moments ($\boldsymbol{\mu}_{\mathrm{ij}}$), which are necessary parameters for calculating V_{ijkl}, and excitation energies of hemes (σ_{ij}) were obtained by time-dependent density functional theory (TDDFT: Gaussian 09) calculations based on structures of hemes having two imidazole axial ligands in a crystal structure of the oxidized MtrC (PDB code: 4LB8). The TDDFT calculations of hemes of reduced MtrC were carried out by changing the total charge of hemes without changing structure of oxidized MtrC. In the TDDFT calculations, B3LYP correlation function was applied. We used the 6-31G* basis set for the hydrogen, carbon, nitrogen and oxygen atoms, the LANL2DZ basis set for iron ion (X. Ming *et al*, *J. Phys. Chem. B*, **2008**, 112, 990−996). In the case of porphyrin analogies, two transitions, whose electric transition dipole moments are degenerate and orthogonal to each other, exist in the Soret band region ($\boldsymbol{\mu}_{\mathrm{ix}}$ and $\boldsymbol{\mu}_{\mathrm{iy}}$ for unit i). Thus, the determinant of purified MtrC should be evaluated for calculating twenty excited-state energies, ε_{n} (n = 1−20), and twenty eigenfunctions, Ψ_{n} (n = 1−20), which is represented by the linear combination of the basis function ϕ_{ij}, which describes unit i (i = 1−10) in the excited state j (j = x, y) as follows.

$$\begin{vmatrix} \sigma_{1x}-\varepsilon & 0 & V_{2x1x} & V_{2y1x} & \cdots & V_{10y1x} \\ 0 & \sigma_{1y}-\varepsilon & V_{2x1y} & V_{2y1y} & \cdots & V_{10y1y} \\ V_{1x2x} & V_{1y2x} & \sigma_{2x}-\varepsilon & 0 & \cdots & V_{10y2x} \\ V_{1x2y} & V_{1y2y} & 0 & \sigma_{2y}-\varepsilon & \cdots & V_{10y2y} \\ \vdots & \vdots & \vdots & \vdots & \ddots & \vdots \\ V_{1x10y} & V_{1y10y} & V_{2x10y} & V_{2y10y} & \cdots & \sigma_{10y}-\varepsilon \end{vmatrix} = 0 \quad (1)$$

Here, σ_{ij} is excitation energy of the heme. Off-diagonal term (V_{ijkl}) is represented under a point-dipole approximation as follows.

$$V_{\mathrm{ijkl}} = \frac{\boldsymbol{\mu}_{\mathrm{ij}} \cdot \boldsymbol{\mu}_{\mathrm{kl}}}{|\mathbf{r}_{\mathrm{ik}}|^3} - 3\frac{(\boldsymbol{\mu}_{\mathrm{ij}} \cdot \mathbf{r}_{\mathrm{ik}})(\boldsymbol{\mu}_{\mathrm{kl}} \cdot \mathbf{r}_{\mathrm{ik}})}{|\mathbf{r}_{\mathrm{ik}}|^5} \quad (2)$$

$\mathbf{r}_{\mathrm{ij}}$ denoted the distance vector between i and j. The eigenvalues and eigenvectors were obtained by diagonalizing the matrix. Rotational strength (R_{n}) is expressed as follows.

$$R_{\mathrm{n}} = \pi \sum_{j=x,y} \sum_{i=1}^{10} \sigma_{\mathrm{ij}} c_{\mathrm{ij}} \boldsymbol{\mu}_{\mathrm{ij}} \cdot \sum_{j=x,y} \sum_{i=1}^{10} c_{\mathrm{ij}} \mathbf{r}_{\mathrm{i}} \times \boldsymbol{\mu}_{\mathrm{ij}} \quad \mathbf{(3)}$$

Supplementary Figures

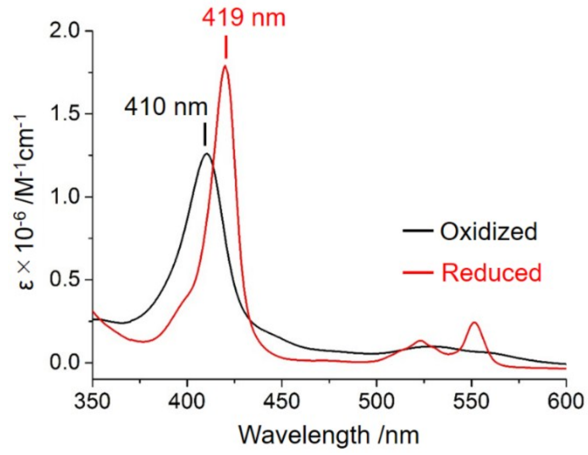

Figure S1. Electronic absorption spectra of purified MtrC. Reduced MtrC is indicated by the red line, and was achieved by the addition of 0.67 mM $Na_2S_2O_4$ to oxidized MtrC, which is depicted by the black line.

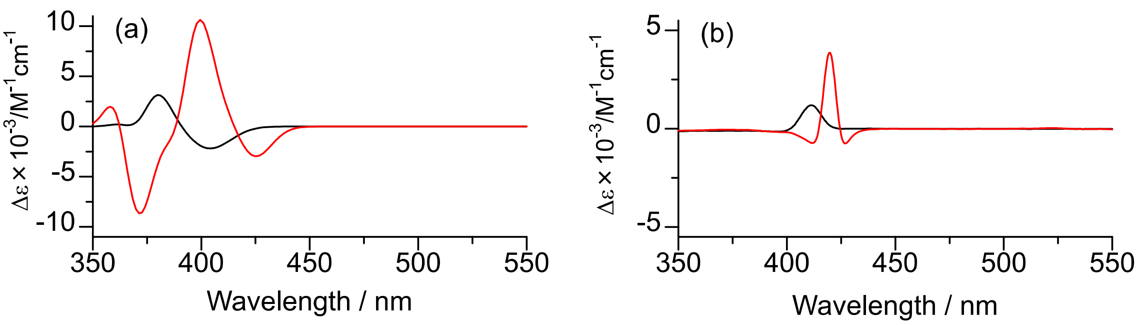

Figure S2. (a) Calculated CD spectra of MtrC (oxidized form: black line, reduced form: red line), and (b) observed CD spectra of purified MtrC (oxidized form: black line, reduced form: red line). It was reproduced that the intensity of positive peak in reduced form was three times larger than that in oxidized form although the intensity of calculated CD was larger than that of observed CD because of the overestimation due to the point-dipole approximation. This indicates that the spectral change from the oxidized state to the reduced state can be explained by the increase of the electric transition dipole moment which enhances CD intensity in reduced MtrC. This is consistent with the fact that molecular extinction coefficient at the Soret band of reduced MtrC is larger than that of oxidized MtrC (Figure S1).

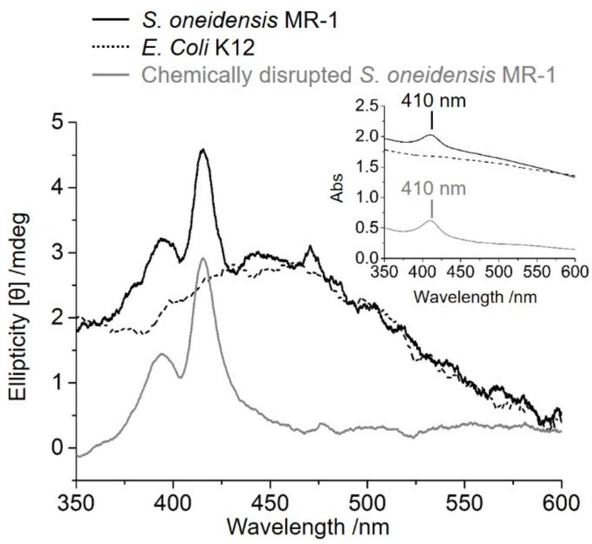

Figure S3. CD spectra of *S. oneidensis* MR-1 (black solid line), *E. coli* K12 (black dotted line), and *S. oneidensis* MR-1 broken by CelLytic B (gray solid line). While broad peak was observed throughout visible light wave range in *S. oneidensis* MR-1, almost identical peak appeared in the cell suspension of *E. coli* and diminished by chemical disruption of cells, indicating that the broad background originates from the light scattering of microbial cell surface. Inset: Absorption spectra obtained simultaneously with CD spectra.

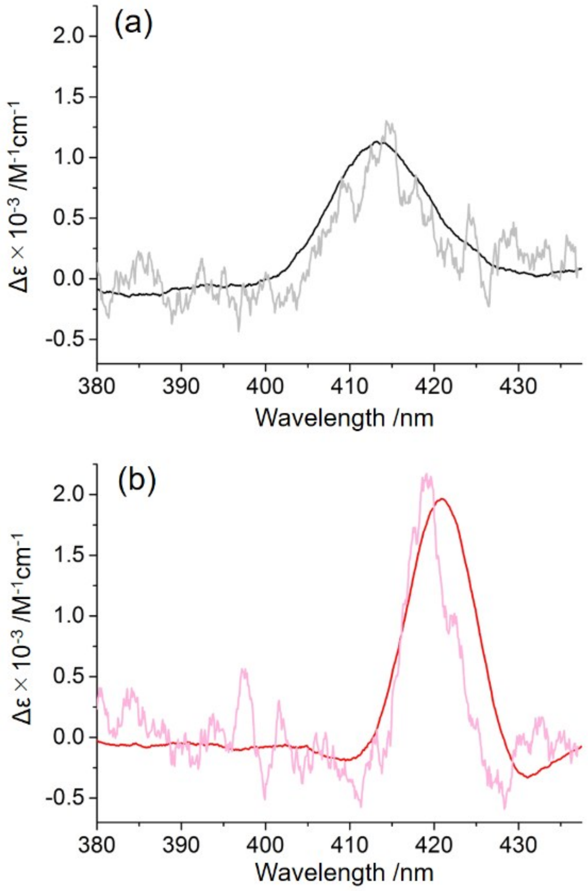

Figure S4. Whole-cell CD difference spectra between WT and *ΔmtrC* strain of *S. oneidensis* MR-1 under aerobic condition in the absence of lactate (oxidized state) (a) and under anaerobic condition in the presence of lactate (reduced state) (b). Black and red lines were obtained with bandwidth as 5.0 nm and OD_{600} as 1.33±0.02. In contrast, when whole-cell CD spectra were measured at OD_{600} = 0.67±0.01 in the identical condition with purified MtrC (1.0 nm bandwidth, 50-time integration), the signal-to-noise ratio was poor as seen in gray and pink lines. To prevent the reduction of MtrC by metabolically generated electrons during measurement in (a), 30 mM fumarate was supplemented (T. Shibanuma *et al, Angew. Chem. Int. Ed.,* **2011**, 50(39), 9137-9140).

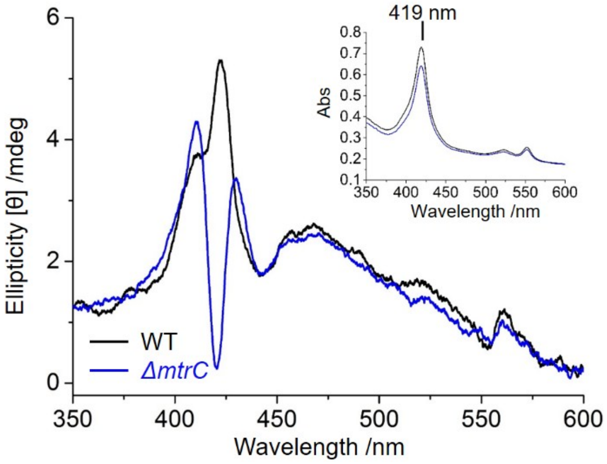

Figure S5. CD spectra of *S. oneidensis* MR-1 wild-type (WT; black line) and mutant strain deficient in the gene encoding MtrC (*ΔmtrC*; blue line) in the presence of 30 mM lactate under anaerobic condition. Inset: Absorption spectra of living *S. oneidensis* MR-1 WT and *ΔmtrC* in diffused transmission mode.

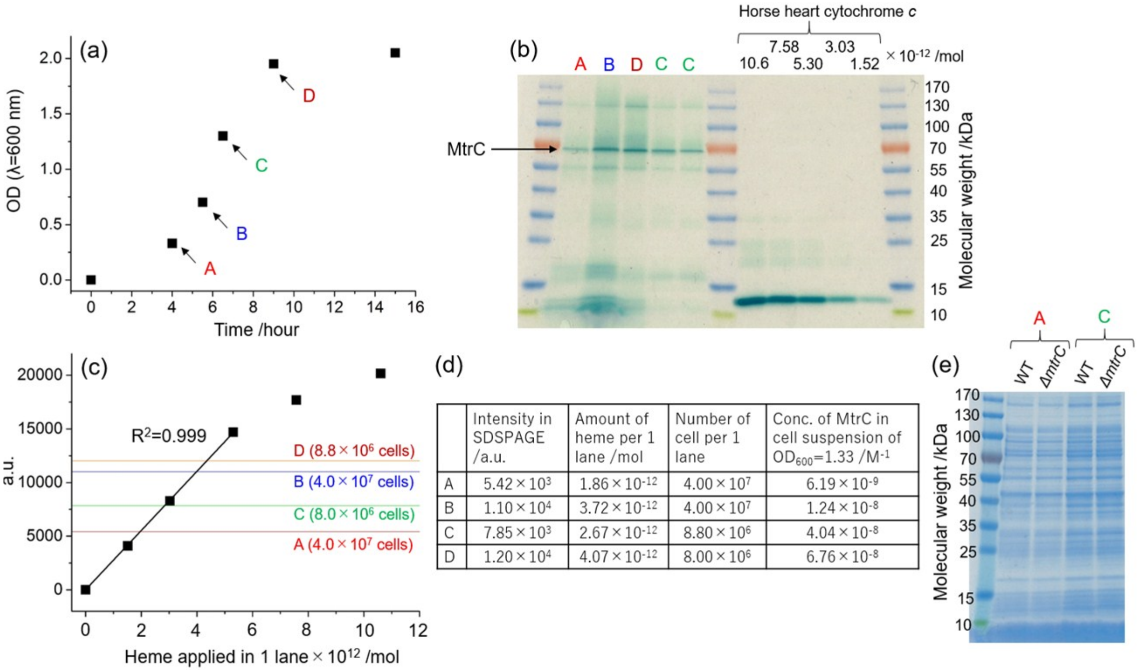

	Intensity in SDSPAGE /a.u.	Amount of heme per 1 lane /mol	Number of cell per 1 lane	Conc. of MtrC in cell suspension of OD_{600}=1.33 /M^{-1}
A	5.42×10^{3}	1.86×10^{-12}	4.00×10^{7}	6.19×10^{-9}
B	1.10×10^{4}	3.72×10^{-12}	4.00×10^{7}	1.24×10^{-8}
C	7.85×10^{3}	2.67×10^{-12}	8.80×10^{6}	4.04×10^{-8}
D	1.20×10^{4}	4.07×10^{-12}	8.00×10^{6}	6.76×10^{-8}

Figure S6. Quantification of MtrC by SDSPAGE. (a) Growth curve of *Shewanella oneidensis* MR-1 during aerobic cultivation. The points from A to D were sampled and subjected to disruption followed by SDSPAGE. (b) SDSPAGE stained by a method specific for heme proteins. The samples from A to D in (a) and horse heart cytochrome *c* are tested. The arrow indicates the band for MtrC, which is confirmed by SDSPAGE for purified of MtrCAB complex from *S. oneidensis* MR-1. Note that the number of *S. oneidensis* MR-1 cells applied to each lane is different among the sample from A to D (A, 4.0×10^{7} cells; B, 4.0×10^{7} cells; C, 8.0×10^{6} cells; D, 8.8×10^{6} cells, assuming that OD_{600} = 1.0 represents 1.0×10^{9} cells per mL) (c) The calibration curve for the intensity of the band for horse heart cytochrome *c* in (b) against the amount of hemes. The intensity was measured by free-software, Image J. (d) Estimation of MtrC concentration in each sample from A to D based on the calibration curve obtained in (c). (e) Comparison of SDSPAGE of wild type (WT) and *ΔmtrC* stained by Coomassie blue. The profile showed little difference between WT and *ΔmtrC*, suggesting that the deletion of *mtrC* gene has scarce impact on gene expression of major proteins other than MtrC.

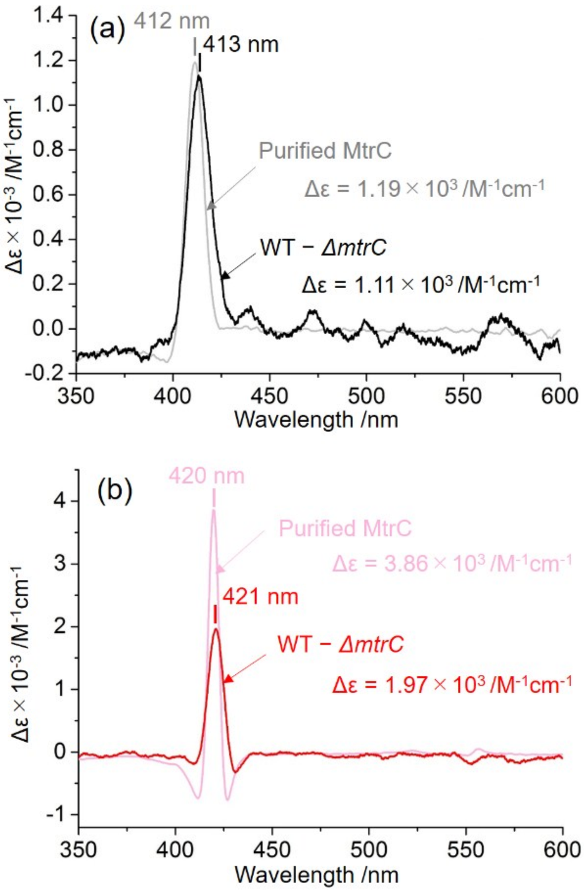

Figure S7. Comparison of CD spectra of purified MtrC and whole-cell CD difference spectra of *S. oneidensis* MR-1 (WT) subtracted by *ΔmtrC* in oxidized state (a) and reduced state (b).

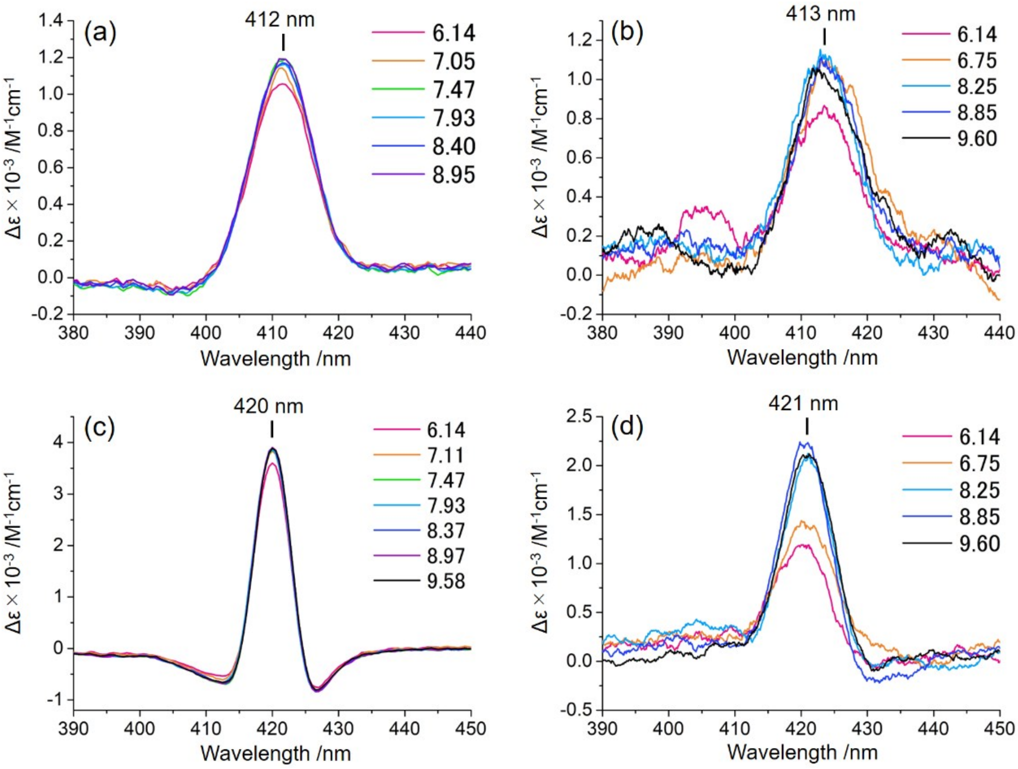

Figure S8. CD spectra of purified MtrC in oxidized state (a) and reduced state (c), and whole-cell CD spectra in oxidized state (b) and reduced state (d) at various pH within the physiological range. The cell suspension were incubated for 10 minutes after adjustment of pH to equilibriate the local pH around native MtrC in whole cell. The effect of pH was immediately appeared at the pH of between 7.4 and 6.8. The sample pH adjusted by NaOH or H_2SO_4 is indicated.

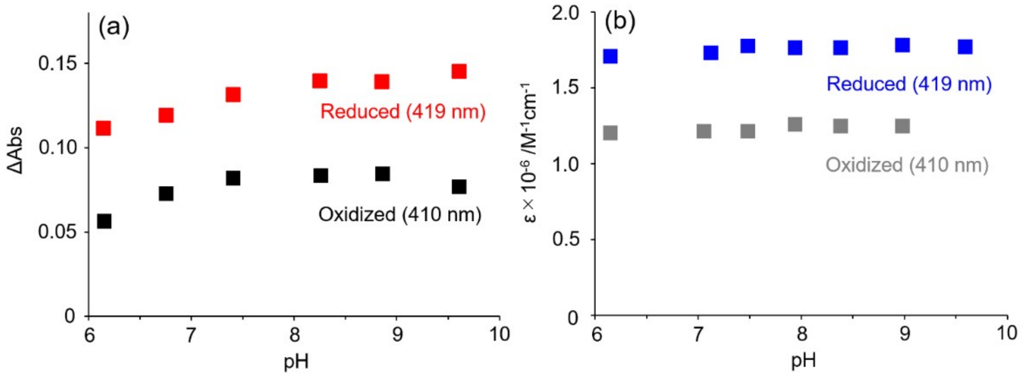

Figure S9. (a) Plots of the Soret ΔAbs between WT and *ΔmtrC* as a function of pH. Black and red plots represent the oxidized (410 nm) and reduced (419 nm) state, respectively. The Soret extinction coefficient change might be assignable to conformation change and local environmental change of hemes as reported previously (Massimo, C. *et al, J. Bio. Chem.* **1997,** 272(40), 24800–248404) (Goto, Y. *et al, Biochemistry* **1993,** 32, 11878–11885). Because the extent of decrease of Δε was larger than that of absorption peak intensity (Figure 3b), the change of electric transition dipole moment among hemes most likely occur (Harada, N. *et al, Circular dichroic spectroscopy: exciton coupling in organic stereochemistry*. Univ Science Books: **1983**.) (Berova, N. *et al, Chem. Soc. Rev.* **2007,** *36* (6), 914–931). Those data indicate that the decrease of Δε reflects the conformation change of hemes in MtrC dependent on pH. (b) Soret extinction coefficient of purified MtrC protein as a function of pH. The blue and gray plots represent the MtrC in reduced state and oxidized state, respectively.

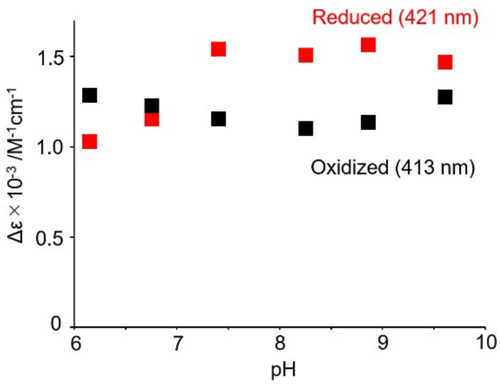

Figure S10. Soret peak intensities in the CD spectra of native reduced MtrC ($\Delta\varepsilon_{(421nm)}$, red) and native oxidized MtrC ($\Delta\varepsilon_{(413nm)}$, black) as a function of pH based on the assumption that the concentratoin of MtrC in whole-cell changes according to the absorption peak intensity in Figure S9. Although the absorption change of MtrC against pH in Figure S9 is most likely assignable to conformational change of heme centers, we examined the Δε with an assumption that concentration of MtrC alters upon pH change. The Δε decrease at pH of between 7.4 and 6.8 was observed, further supporting the conclusion of Figure 3b that the pH induced the heme conformational change.

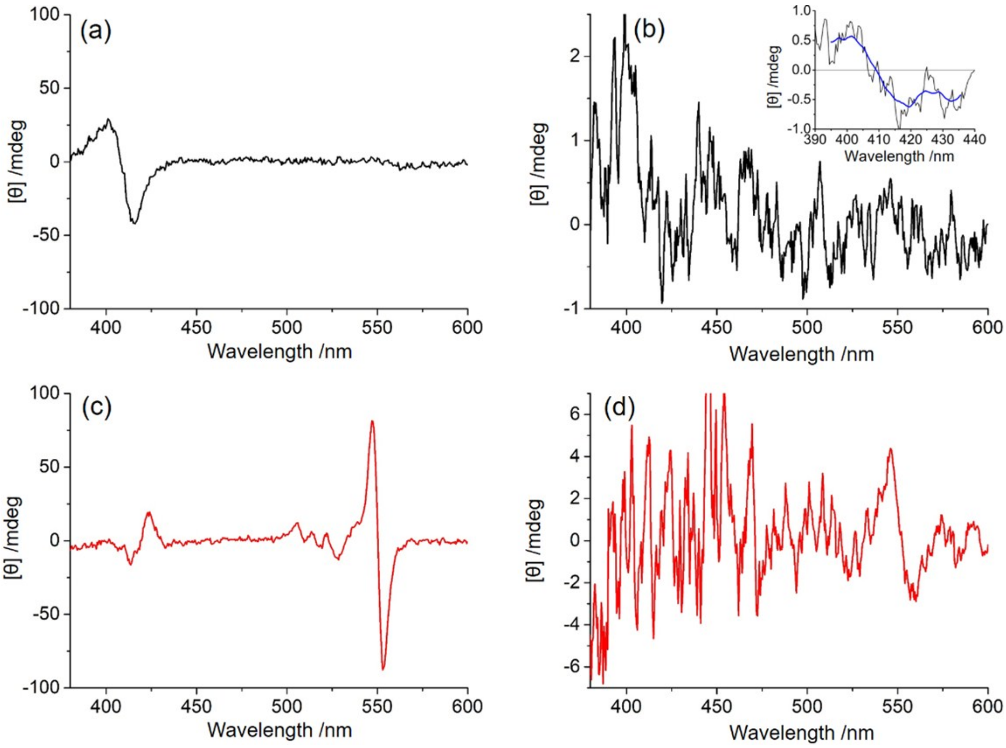

Figure S11. Magnetic circular dichroism (MCD) spectra of purified MtrC in oxidized state (a) and reduced state (c), and MCD difference spectra between WT and *ΔmtrC* in the absence of (oxidized state) (b) and the presence of lactate (reduced state) (d). 0.89 μM MtrC was used in (a) and (c), and the cell density was adjusted to OD_{600} = 0.60 ± 0.01 in (b) and 1.33 ± 0.02 in (d) prior to the measurement. Spectra were integrated 50 times in (b) and (d), and 200 times in inset (b), of which smoothed line is represented as blue. For maintaining oxidized state of MtrC during integration in (b), 50 mM fumarate was supplemented as reported (T. Shibanuma *et al, Angew. Chem. Int. Ed.,* **2011**, 50(39), 9137-9140). In oxidized state, both the purified MtrC and whole-cell showed a dispersion type Faraday A term at Soret region, which is consistent with the feature of two histidine residues as axial ligands reported as oxidized crystal structure (Edwards M. J., *et al. Sci. Rep.,* **2015**, 5, 11677). After reduction, Faraday A term was observed with center wave length around 550 nm in both the purified and native MtrC, which is a characteristic of the low-spin ferrous state (Field. S. J., *et al, J. Biol. Chem.,* **2000**, 275, 8515-8522). Notably, this spectral tendency is consistent with reduced heme with histidine residues as axial ligands, e.g. *c*-Cyt isolated from *Shewanella frigidimarina* NCIMB400 (Field. S. J., *et al, J. Biol. Chem.,* **2000**, 275, 8515-8522). Furthermore, the positive CD signal observed at the Soret band of reduced MtrC in whole-cell is similar to the CD spectra of c_3-Cyt purified from *Shewanella oneidensis* TSP-C, which has two histidine residues as axial ligands (Y. Takayama *et al, Biochemistry,* **2006**, 45, 3163-3169). Thus, these MCD and CD spectra suggest that the reduced MtrC in whole-cell maintains the coordination of bis-histidine.

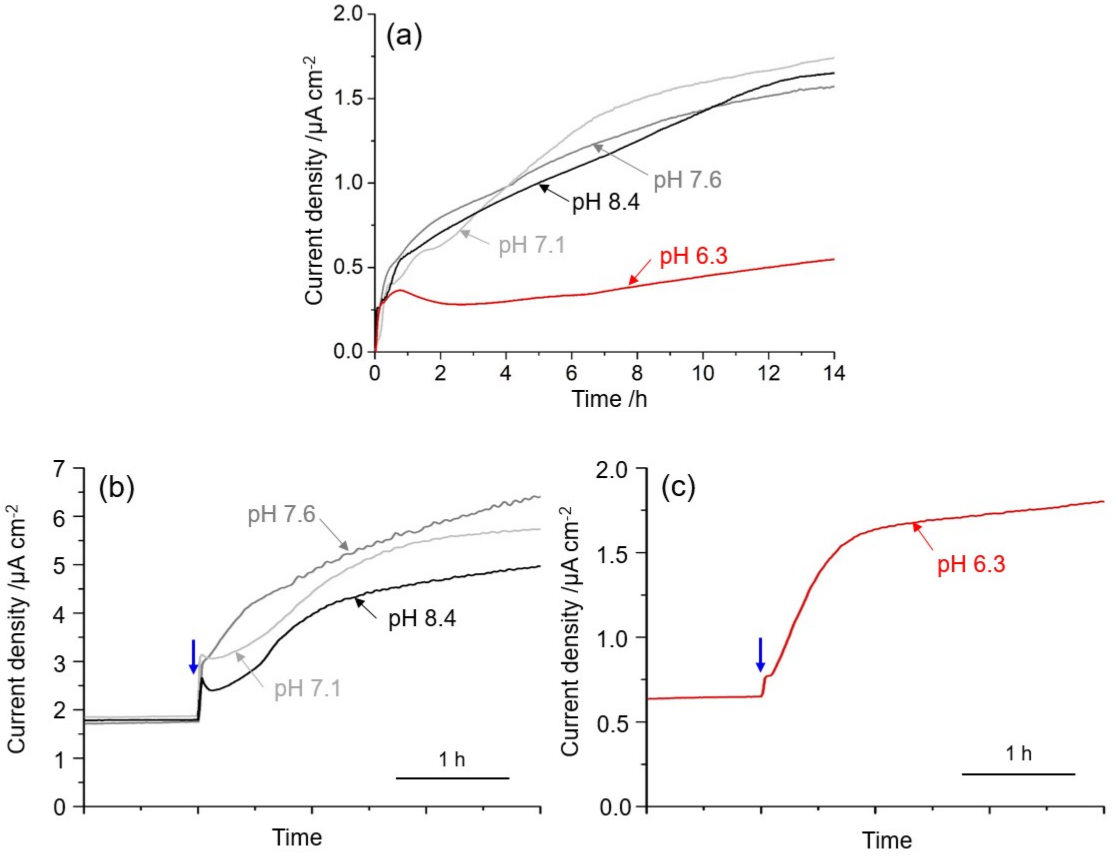

Figure S12. (a) Time course for the current production from *S. oneidensis* MR-1 cells in the presence of 10 mM lactate on an ITO electrode under +0.4 V (versus SHE) at various physiological pH. The cell suspension was injected into the electrochemical reactors at t = 0. The pH of the medium in the electrochemical reactors is indicated. The same tendency was observed in at least two individual experiments. (b), (c) Effect of 2.0 µM flavin mononucleotide (FMN) addition into the same electrochemical reactors with (a) on the current production. Blue arrows indicate the timing of FMN addition. The addition of FMN drastically increased the current production in all the condition, demonstrating that the electron transport via outer-membrane *c*-type cytochrome complex (OM *c*-Cyts) limits the current production as reported (Y. Tokunou, *et al, J. Vis. Exp.*, **2018**, 134, 57584) (J. Saito, *et al, Electrochim. Acta,* **2016**, 216, 261-265).

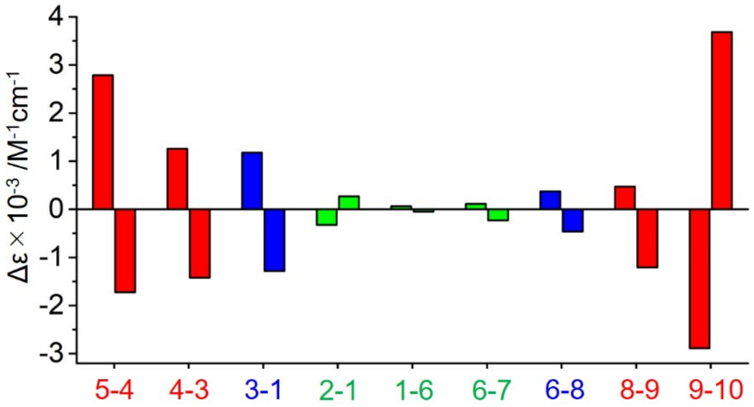

Figure S13. Calculated peak CD intensities based on the exciton interaction between adjacent heme pairs in the reduced MtrC. The bars are colored according to the inter-hemes orientation: stacked in red, T-shaped in blue, and coplanar in green. The dispersion-type peak CD signals are devided into shorter and longer wavelength parts, represented by left-side and right-side bars, respectively. In each CD calculation, the only off-diagonal term concerning the adjacent heme was considered.

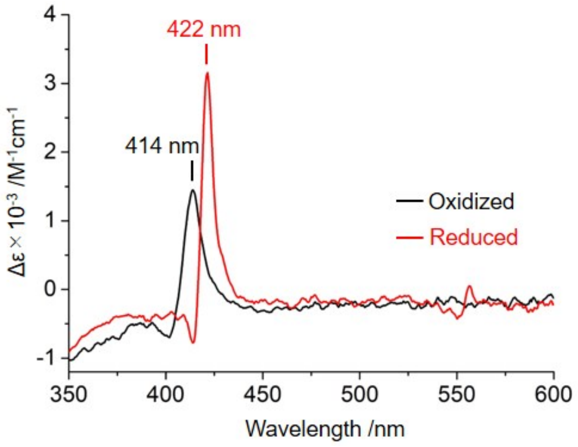

Figure S14. CD spectra of MtrCAB complex, which was purified as reported previously (Daniel E. Ross *et al, Appl. Environ. Microbiol.*, **2007**, 73(18), 5797-5808). Reduced MtrCAB (red line) was achieved by the addition of 1.0 mM $Na_2S_2O_4$ to oxidized MtrCAB (black line).
